# Multilevel Estimation of the Relative Impacts of Social Determinants on Income-Related Health Inequalities in Urban Canada: Protocol for the Canadian Social Determinants Urban Laboratory

**DOI:** 10.2196/71929

**Published:** 2025-11-28

**Authors:** Charles Plante, Suvadra Datta Gupta, Thilina Bandara, Daniel Beland, Christine Blaser, Cheryl A Camillo, Eileen de Villa, Daniel Dutton, Daniel Fuller, Jasmine Hasselback, Lisa Marie Lix, Anousheh Marouzi, Nazeem Muhajarine, Geranda Notten, Bill Reimer, Michael Wolfson, Marisa Young, Daniel Yupanqui Concha, Cory Neudorf

**Affiliations:** 1 Saskatchewan Health Authority Saskatoon, SK Canada; 2 Department of Community Health and Epidemiology University of Saskatchewan Saskatoon, SK Canada; 3 School of Public Health University of Saskatchewan Saskatoon, SK Canada; 4 Department of Political Science McGill University Montreal, QC Canada; 5 Department of Social and Preventive Medicine, School of Public Health University of Montreal Montreal, QC Canada; 6 Johnson Shoyama Graduate School of Public Policy University of Regina Regina, SK Canada; 7 Toronto Public Health Toronto, ON Canada; 8 Department of Community Health and Epidemiology Dalhousie University Halifax, NS Canada; 9 Department of Community Health Sciences University of Manitoba Winnipeg, MB Canada; 10 Research Department Saskatchewan Health Authority Saskatoon, SK Canada; 11 Graduate School of Public and International Affairs University of Ottawa Ottawa, ON Canada; 12 Department of Sociology and Anthropology Concordia University Montreal, ON Canada; 13 School of Epidemiology and Public Health University of Ottawa Ottawa, ON Canada; 14 Department of Sociology McMaster University Hamilton, ON Canada; 15 Department of Agricultural and Resource Economics University of Saskatchewan Saskatoon, SK Canada

**Keywords:** social determinants of health, health inequity, data linkage, decomposition analysis, multilevel model

## Abstract

**Background:**

Two decades of research have highlighted persistent income-related health inequities in Canada across municipal, provincial, and national levels. While there is broad consensus among researchers, advocates, and health professionals that social determinants are the primary drivers of health, the empirical foundation supporting this remains relatively limited. A current renaissance in health system data access offers an opportunity to assess the multilevel impact of social factors on health inequalities, yet this potential remains underused.

**Objective:**

This project aims to examine how social, economic, and political conditions shape health inequalities and investigate how structural and intermediate determinants explain disparities across national, provincial, city, neighborhood, and individual levels.

**Methods:**

We will create the Canadian Social Determinants Urban Laboratory (CSDUL), a multilevel, longitudinal, virtual data environment that integrates 15 existing databases from Statistics Canada, the Canadian Institute for Health Information, the Canadian Urban Environmental Health Research Consortium, and DMTI Spatial. Guided by the World Health Organization social determinants of health framework, CSDUL will initially cover 2011 to 2015 due to data completeness and expand as additional years become available. CSDUL builds on Statistics Canada’s Canadian Population Health Survey and will link survey data to administrative and health records, including hospital discharges, ambulatory care, mortality, cancer registries, and longitudinal tax files. Area-level indicators will be added using historical postal codes and geospatial boundaries. Organized through a hub-and-node model, CSDUL includes a central hub and 5 research nodes. We will develop and validate area-based indicators to study social determinants at micro (individual), meso (neighborhood, city, and province), and macro (national) levels. A core deliverable is to assess the strengths and limitations of survey and administrative data for health research and derive variables accordingly. After developing CSDUL, we will replicate World Health Organization Regional Office for Europe income-related health inequality analysis for urban Canada and analyze the impact of social determinants on outcomes. We will apply a 2-fold Oaxaca-Blinder decomposition between the lowest and highest urban income quintiles. A major strength of CSDUL is its capacity to analyze how diverse determinants shape health across subgroups (eg, gender), identifying key drivers of health outcomes.

**Results:**

The indicators to be used in CSDUL are being developed and validated by the contributing nodes. In collaboration with node 3, we are constructing measures of social capital using DMTI Spatial Points of Interest data. A prototype version of CSDUL incorporating a limited set of indicators has been developed in Statistics Canada’s Research Data Centre. We anticipate receiving the finalized indicators from the nodes by August 2025 to September 2025 and aim to complete the decomposition analysis by December 2025.

**Conclusions:**

Multisectoral interventions are most effective when they are customized to meet the unique needs of specific subpopulations using robust and multilevel data sources such as CSDUL.

**International Registered Report Identifier (IRRID):**

DERR1-10.2196/71929

## Introduction

### Background

Despite a mounting consensus among researchers, advocates, and health professionals that social determinants are the primary drivers of the health of Canadians, the empirical basis for this claim remains thin. Studies from several European countries have shown that income and social security are primary drivers, with social determinants of health (SDOHs) explaining up to 89% of the variation in self-reported health in these countries [1]. A similar trend is observed in the United States, where socioeconomic factors tend to be the strongest predictors of health outcomes [2]. One of the very few flagship reports describing health disparities across Canadian cities, *Reducing the Gaps in Health: A Focus on Socio-Economic Status in Canada*, championed by the Urban Public Health Network (UPHN), found that income-related health inequalities have remained largely unchanged since the early 2000s and vary considerably between cities and indicators [3-5]. Many of these inequalities persist at municipal, provincial, and national levels and are direct consequences of individuals’ socioeconomic disadvantages [3,6]. As SDOHs are many and their interactions are complex [7,8], a more in-depth account of how social determinants impact health is needed to identify the highest-impact targets for interventions that can reduce these inequalities and improve health outcomes.

Considerable data are required to measure different aspects of the SDOHs, and these data have to be combined, motivating calls for more significant investments in linkages between health and social data [9-12]. During the past 2 decades, Canadian research data access centers have developed a new way of conducting health and social research using nationally representative linked surveys and administrative data [13]. However, despite offering a significant amount of data on the various SDOHs, the advanced knowledge of these data sources that is needed to work with them is limited among researchers and academics [14]. Considerable disparities also exist in the capacity of local health units to survey regional differences in population health and health equity, with some units having no capacity at all [15,16], and becoming familiar with large and complex data sources such as those in the Research Data Centres (RDCs) is costly. Even among units that do have the capacity to make these investments, work tends to be carried out in isolation, and no national standards exist to ensure that research is carried out in ways that support comparative learning [17-19].

This paper presents an innovative protocol for the world’s first fully integrated virtual, multilevel, and longitudinal social determinant laboratory environment, entitled Canadian Social Determinants Urban Laboratory (CSDUL). CSDUL aims to facilitate multilevel statistical analyses of how social and structural determinants of health impact the overall health of Canadians**―**achieved by creating a set of programs, algorithms (statistical codes and syntaxes), and data components accumulating information from at least 15 primary data sources within the secure research environment of the RDCs. The project will further use CSDUL to replicate the decomposition analysis of income-related inequalities in self-reported health carried out by the World Health Organization (WHO) Regional Office for Europe (WHO/Europe) [20] for urban Canada. Decomposition analysis is a method used to investigate changes in specific indicators by breaking them down into various contributing factors, enabling us to identify the underlying drivers of these changes [19].

In short, this project will allow us to determine how much of the variation in income-related health inequalities in Canada can be attributed to the various SDOHs (ie, report on the relative impact of social determinants on health inequities). In addition to replicating previous studies that have illustrated the contribution of SDOHs in determining health status, the CSDUL environment will allow us to examine how different categories of SDOHs operate on health at different levels and bridge the gap between the WHO’s conceptual and action frameworks. Our research will adopt a slightly modified version of the WHO *Conceptual Framework for Action on the Social Determinants of Health* [21]. The WHO SDOH framework illustrates how social, economic, and political mechanisms give rise to environmental and socioeconomic conditions and how these, in turn, shape access to important determinants of health status [21]. One of the innovations of the SDOH framework is that it also conceptualizes the health system as a social determinant. However, the WHO’s separate but complementary framework for action on tackling SDOH inequalities identifies 4 levels of *mechanisms*—global, macro, meso, and micro—that motivate different kinds of policy actions to improve population health and reduce inequalities [21].

### Objectives

The specific research aims of this project will be to work with Statistics Canada’s integrated Canadian Population Health Survey (CPHS) data [22] and other leading social determinant data to (1) develop and distribute the world’s first fully integrated virtual, multilevel, and longitudinal social determinant laboratory environment, which we will call CSDUL; and (2) replicate the decomposition analysis of income-related inequalities in self-reported health carried out by the WHO/Europe for urban Canada and report on the relative impact of social determinants.

## Methods

### Data

#### Overview

The backbone of CSDUL’s data structure will be Statistics Canada’s CPHS [22], which researchers can access via RDC programs. The CPHS consists of linked survey, administrative, and registry data among the Canadian Community Health Survey (CCHS) [23], the Discharge Abstract Database [24], the National Ambulatory Care Reporting System [25], the Canadian Vital Statistics–Death database [26], the Canadian Cancer Registry [27], and 20 years of Canada Revenue Agency personal and familial tax records [22]. These data will be merged with area-level indicators using historical postal codes in the CPHS, Canada Post’s Postal Code Conversion File Plus [28], and census boundary files [29] provided by Statistics Canada. Additional data sources include the Canadian Classification of Functions of Government [30], data from the Canadian Urban Environmental Health Research Consortium [31], DMTI Spatial Enhanced Points of Interest [32], and Statistics Canada’s Canadian Social Environment Typology (CanSET) [33]. Table 1 provides a brief description of the datasets. The period with existing data for all these sources is 2011 to 2017. This will be the initial observation window for CSDUL, but we aim to extend it in future studies as new data become available. The CCHS samples approximately 65,000 Canadians each year, and we will add new years as they become available, resulting in an overall N of approximately half a million individual Canadians and a number of health events (eg, hospitalizations or deaths) that is an order of magnitude greater.

**Table 1:** Description of the datasets.

Data source	Description
CCHS^a^	The CCHS is a cross-sectional self-reported survey providing provincial and subprovincial (health region or combined health regions)–level estimates of health status, health care use, and health determinants [23]. The CCHS comprises 2 types of surveys: an annual component survey on general health administered to all the sample and focused surveys on specific health topics from which the provinces and territories can opt out [34].
CPHS^b^	The CPHS links various Canadian datasets, including the CCHS annual and focus content, CVSD^c^, DAD^d^, NACRS^e^, CCR^f^, and 20 years of personal and familial tax records, providing researchers with analytical datasets that contain integrated data on mortality, hospitalization, historical postal codes, cancer, tax data, and censuses [22].
CanSET^g^	A qualitative measure of the social environment in Canadian cities entitled CanSET was created using 30 socioeconomic, demographic, and ethnocultural variables derived from the 2016 census [33]. Aggregated at the dissemination area level, CanSET clusters portray a comprehensive picture of urban social and health inequalities in Canada [33].
CVSD^c^	The CVSD is a comprehensive, cross-sectional census that collects demographic and medical data, including causes of death classified according to the World Health Organization’s ICD^h^, from all provincial and territorial vital statistics registries on a monthly and annual basis [26].
CCR^f^	The CCR is a national population-based registry that provides person-level information on the type and characteristics of each new primary cancer diagnosed among Canadian residents since 1992 [27]. It includes information provided by the 13 Canadian PTCRs^i^ except Quebec (since 2018) and Nova Scotia (since 2020) [27].
Canadian Census of Population	The Canadian Census of Population is conducted every 5 y and includes a short survey administered to every Canadian and a long-format survey administered to roughly 25% of the subsample (replaced by the National Household Survey in 2011) [35]. The census database contains detailed demographic and socioeconomic data on the Canadian population living in its provinces, territories, and municipal areas [35].
CCOFOG^j^	The CCOFOG classifies areas of government spending according to international standards, allowing for comparisons of government expenditures across jurisdictions [30]. Covering the years between 2008 and 2020, the CCOFOG illustrates the total expenditure by the various government units (federal, provincial and territorial, and local) in 10 categories: general public services, defense, public order and safety, economic affairs, environmental protection, housing and community amenities, health, recreation, culture and religion, education, and social protection [36].
CANUE^k^	The CANUE provides a comprehensive and easily accessible set of national urban exposure metrics from 1980 onward [31]. It has assembled several datasets capturing built and natural environmental exposures at the postal code level across the country [31]. This includes variables describing greenness, proximity to water bodies, proximity to roads, walkability, noise, light and air pollution, and gentrification [31].
Census boundary files	Statistics Canada releases census boundary files each census year in 3 types of geographic areas: administrative boundaries (eg, provinces and territories, health regions, and federal electoral districts), statistical boundaries (eg, census metropolitan areas and census agglomerations, census tracts, and dissemination areas), and nonstandard boundaries (eg, population ecumene) [29]. In addition, boundary files for the intercensal years containing only CSD^l^ boundaries are also released, and CSDUL^m^ will use both of these types of files to link multiple datasets [37].
CIHI^n^	The CIHI collects and reports comparable pan-Canadian data on various aspects of the health system, such as health care access, appropriateness, and quality at the health region level on the Your Health System online portal [38]. We will use this portal to create a suite of ready-made and documented area-level health system indicators for inclusion in CSDUL.
DAD^d^	The DAD^d^ is collected and reported by the CIHI, containing detailed administrative, clinical, and demographic information on hospital discharges, including deaths, sign-outs, and transfers, at the provincial and territorial levels except for Quebec [24]. This database can be used to calculate various population health indicators, providing important insights into regional health inequities, including hospitalizations for opioid overdoses, alcohol-related harm, attempted suicide, and various injuries [24].
DMTI Spatial EPOI^o^	The EPOI file, produced by DMTI Spatial Inc, is a national database that contains >1 million business and recreational locations across Canada [32]. This resource allows users to view and analyze point-of-interest data within a specific geographic area, facilitating applications such as market research and business analysis [32]. We are measuring social capital based on this data source.
NACRS^e^	The NACRS^e^ data are sourced directly from participating facilities, regional health authorities, or health ministries [25]. It includes information on hospital-based and community-based ambulatory care services (eg, day surgery, outpatient services, and emergency department services) [25].
CRA^p^ tax records	CRA tax records contain information about taxes, benefits, and related details for most Canadian provinces and territories, such as income tax records [22].
PCCF+^q^	The PCCF+ file serves as a connection among 6-digit postal codes from the CPC^r^, standard census geographic areas (including dissemination areas, census subdivisions, and census tracts) created by Statistics Canada, and supplementary administrative areas and neighborhood income quintiles [28].

^a^CCHS: Canadian Community Health Survey.

^b^CPHS: Canadian Population Health Survey.

^c^CVSD; Canadian Vital Statistics–Death database.

^d^DAD: Discharge Abstract Database.

^e^NACRS: National Ambulatory Care Reporting System.

^f^CCR: Canadian Cancer Registry.

^g^CanSET: Canadian Social Environment Typology.

^h^ICD: International Statistical Classification of Diseases and Related Health Problems.

^i^PTCR: provincial and territorial cancer registry.

^j^CCOFOG: Canadian Classification of Functions of Government.

^k^CANUE: Canadian Urban Environmental Health Research Consortium.

^l^CSD: census subdivision.

^m^CSDUL: Canadian Social Determinants Urban Laboratory.

^n^CIHI: Canadian Institute for Health Information.

^o^EPOI: Enhanced Points of Interest.

^p^CRA: Canada Revenue Agency.

^q^PCCF+: Postal Code Conversion File Plus.

^r^CPC: Canada Post Corporation.

Furthermore, at this time, we will restrict CSDUL to Canada’s cities and towns because our aim is to estimate a fully articulated model of the SDOHs, and these factors are not as well understood or measured in rural and remote regions [39-41]. We will classify communities as cities and towns if they qualify as census agglomerations or census metropolitan areas—that is, communities with a core population of at least 10,000 [42]; these entities can be made up of multiple municipalities (eg, the census metropolitan area of Montreal includes Laval, among others). This was the same approach used by Statistics Canada in creating CanSET [33].

In their raw form, most of the databases that will be included in CSDUL are not user-friendly and not readily usable for health analysis. Thus, many of the primary research deliverables of this project will be to critically analyze and document the strengths and limitations of these databases for supporting health research and create and validate the best methods for deriving variables from them. By combining all these data elements, our project will create a multilevel and longitudinal virtual laboratory environment that can be used to examine the operation of SDOHs at micro, meso, and macro levels and study income-related health inequalities. This approach builds on previous studies that could only demonstrate the contributions of SDOHs to health across a limited subset of the WHO SDOH framework elements [2,43].

**Figure 1:**
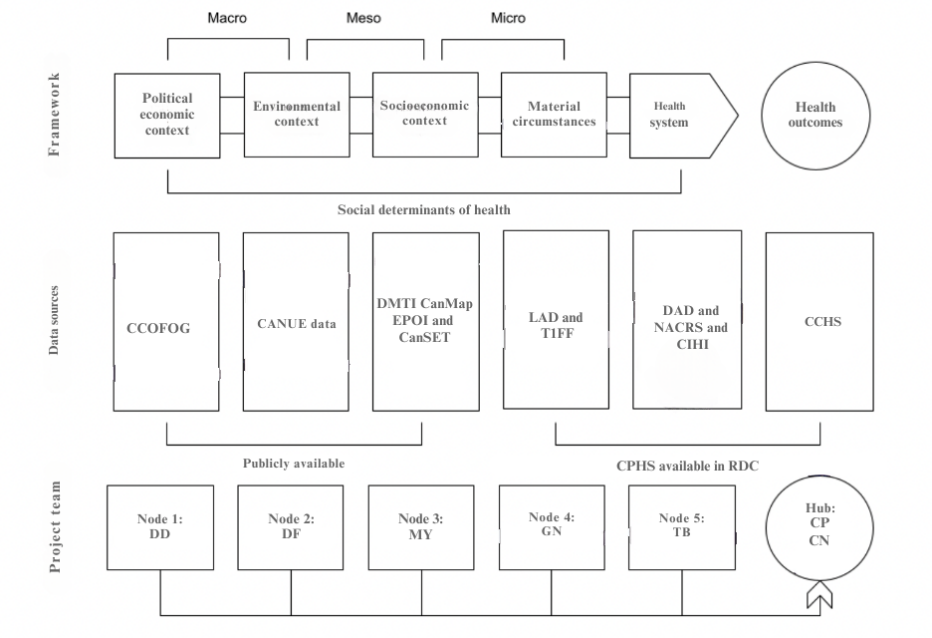
Stylized overview of the Canadian Social Determinants Urban Laboratory, its data structure, and its organizing nodes. CanSET: Canadian Social Environment Typology; CANUE: Canadian Urban Environmental Health Research Consortium; CCHS: Canadian Community Health Survey; CCOFOG: Canadian Classification of Functions of Government; CIHI: Canadian Institute for Health Information; CPHS: Canadian Population Health Survey; DAD: Discharge Abstract Database; EPOI: Enhanced Points of Interest; LAD: Longitudinal Administrative Databank; NACRS: National Ambulatory Care Reporting System; RDC: Research Data Centre; T1FF: T1 Family File.

The development of CSDUL will be organized using a hub and multidisciplinary node teams across Canada and organized around the WHO SDOH framework. Figure 1 provides an overview of the CSDUL project and its mappings between data sources and hub and node activities.

#### Outcome Variable

Self-reported health will be the primary outcome variable of interest for the decomposition analysis. Drawing on validation work by our team using the Health Utilities Index [44,45], we will categorize self-reported health based on whether respondents reported having health that was at least better than *fair*. Self-rated health is a widely used indicator of overall health status, and multiple studies have validated its usefulness as a subjective proxy for a number of objective health measures [46-49].

#### Predictor Variables

**Figure 2:**
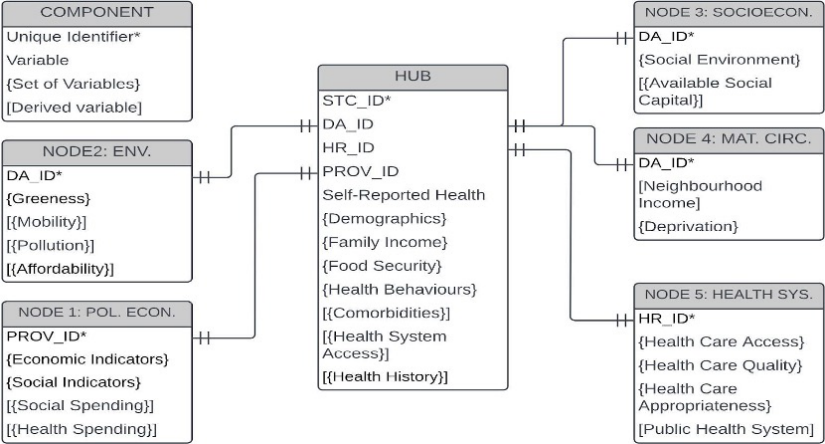
Description of the Canadian Social Determinants Urban Laboratory data components and their relationships. DA_ID: dissemination area identification number; Env.: environmental context; Health syst.: health system; HR_ID: health region identification number; Mat. circ.: material circumstances; Pol. econ.: political and economic context; PROV_ID: province identification number; Socioecon.: socioeconomic context; STC_ID: Statistics Canada identifier.

Each node will contribute tables of predictor variables to the decomposition analysis based on their work on developing CSDUL. To achieve this, working group nodes will conceptualize, operationalize, and link constructs from the WHO conceptual and action social determinant frameworks. Hub individual-level variables will include demographic variables (eg, gender and age), family income, health behaviors [50], health comorbidities [51,52], health system access [38], and health history [53,54]. Political and economic context variables will include economic indicators (eg, macroeconomic policies and Gini coefficient), social indicators (eg, policies on social welfare and land and housing), social spending [55], and health spending [55] at the health region and provincial levels. Environmental context variables will include green space [56], walkability [57], gentrification [58], urban sprawl [59], and air pollution [60] at the dissemination area level in urban areas. Socioeconomic context variables will include the social environment, such as stress and different types of available social capital, including what Klinenberg [61] refers to as *social infrastructure*. Material circumstances will include area-based income and deprivation. Health system indicators will include health care access, health care quality, health care appropriateness, and public health systems. Figure 2 provides an overview of the planned construction of the CSDUL environment, its elements, and its variables.

### CSDUL Development

#### Overview

A central hub operating out of the UPHN Research Group and based at the University of Saskatchewan (hereinafter referred to as the Hub) will assemble data in collaboration with partners, combining them with individual-level indicators of health behavior, history, system access, system use, and outcomes in the CPHS. The Hub will also coordinate the project and ensure that the primary objectives are met. Under the Hub, CSDUL will have 5 project nodes led by different investigators, who will create and validate area-based indicators at the macro and meso levels that will be integrated with CSDUL and the decomposition analysis (objective 2). The nodes are political and economic context (node 1), environmental context (node 2), socioeconomic context (node 3), material circumstances (node 4), and health systems (node 5). In addition, the Hub will assemble and disseminate this virtual environment, which will operate on individual-level data within Statistics Canada’s RDC. The project will be advised by a senior advisory group composed of experts in the field of population health and secondary data development and use. In addition, CSDUL will be advised by a knowledge user group operating as part of the UPHN’s unique Integrated Knowledge Translation Collaborative [62], which brings together academic researchers, national data stewards, and local public health practitioners eager to advance measures and tools that can be used for effective local-level evidence-based decision-making.

#### Node 1: Political and Economic Context

The primary objective of this node will be to study and document the effect of macroeconomic factors and government policy on health outcomes using the new Canadian Classification of Functions of Government data [36]. Statistics Canada has recently updated its Consolidated Government Revenue and Expenditures tables to align with international government revenue and spending practices. This update brings a significant amount of detail, particularly in the areas of health and social spending. This node will create a simplified version of these data (spanning 2008 to 2018) and a set of guidelines for CSDUL. Several macro-level variables from Statistics Canada’s *The Daily* indicator series (eg, Gini coefficients, gross domestic product, and unemployment rate) will be included.

#### Node 2: Environmental Context

Node 2 aims to measure the impacts of environmental factors (ie, walkability, bikeability, food environments, transit accessibility, gentrification, and housing affordability) on health outcomes in Canada’s cities since 2010. The measures already developed are walkability, urban sprawl, bikeability, food environments, transit accessibility, and gentrification. It will also create new neighborhood-level indicators of environmental context using the Canadian Urban Environmental Health Research Consortium data specifically related to housing affordability, as well as validate these measures. CSDUL will be one of the first large-scale validation studies explaining how environmental factors can be combined in population health research with population health outcomes, including self-reported health.

#### Node 3: Socioeconomic Context

Node 3 will expand on the existing theoretical and methodological work conducted on the area-based available social capital in rural communities in Canada [63,64], classifying it into 4 normative types: market, bureaucratic, communal, and associative for urban Canada. This work will be expanded by integrating recent approaches to capturing the social infrastructure in residential areas [61,65,66]. This node will also make use of the UPHN work with the Health Analysis Division of Statistics Canada, which produced a new *qualitative* measure of the social environment**―**the CanSET [33]. The key purpose of node 3 is to investigate the connection between the availability of family-friendly resources from DMTI Spatial (see the study by Young et al [65] and Young and Singh [66] for an overview) and health outcomes using data from the CCHS. Node 3 will also use leading indicators of population health in Canadian cities starting from 2010 from DMTI Spatial and other sources.

#### Node 4: Material Circumstances

The primary objectives of node 4 will be to develop and support the distribution of improved dissemination area–level median after-tax income measures for the CSDUL project and the decomposition analysis. In Canada, income-related health inequalities at the area level have traditionally been measured using income data collected once a year by the census. However, in recent times, social scientists have preferred to use median after-tax income as a more accurate measure of disposable income [67,68]. Canada also has a Longitudinal Administrative Databank that provides longitudinal data on income at the dissemination area level since 1982, but it has not been widely used in health science research. Node 4 extends the use and distribution of a new after-tax income area-level indicator based on the Longitudinal Administrative Databank. It aims to assess the consequences of implementing a new indicator to explore health inequalities associated with income and study how our preference for area-level income or deprivation impacts our research on health inequalities.

#### Node 5: Health System

Node 5 research activities will focus on developing and deploying a suite of area-level (primarily at the health region level) health system indicators to quantify system-level access and quality of care that can be integrated with CSDUL [62,69]. This will involve deriving health system indicators that are available at the subprovincial level and identifying and assessing individual-level indicators of health system access and use from the CCHS.

#### Senior Advisory Group and Knowledge User Group

**Figure 3:**
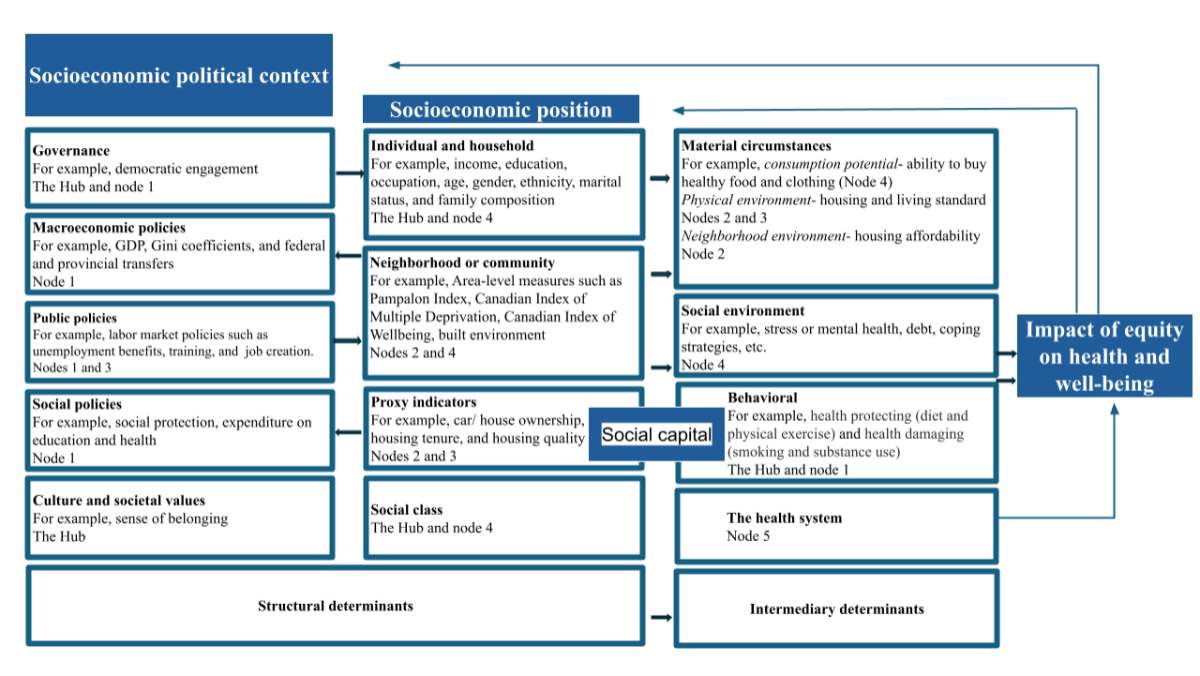
Operationalizing the social determinant of health framework. CCHS: Canadian Community Health Survey; CIW: Canadian Index of Wellbeing; GDP: gross domestic product.

The knowledge user group and the senior advisory group, integral parts of the UPHN Integrated Knowledge Translation Collaborative around the CSDUL project, are composed of the UPHN membership, which includes the country’s top public health medical leadership and the country’s top experts in population health research, respectively. These 2 groups will receive regular updates from the Hub and will provide valuable advice on the design and dissemination of CSDUL related to their areas of expertise and advanced experience. They will also play a key role in facilitating knowledge mobilization within their organizations and among their affiliate health leadership agencies. Figure 3 shows how we plan to operationalize the WHO SDOH framework.

### Decomposition Analysis

Our study will follow the WHO/Europe [20] and use a *2-fold* Oaxaca-Blinder decomposition [70-72] to identify the primary drivers of self-reported health. We are mindful that the CCHS has been redesigned multiple times (most recently in 2015); we will follow best practices [73] and only work with variables that have been measured in the same way over time as we have done in previous work [74]. As was done by the WHO/Europe, we will also report on the distribution of social determinants and health outcomes in the cities and towns we include and replicate our analysis for additional self-rated outcomes**―**mental health and life satisfaction.

The decomposition analysis will be conducted as follows. If *y*_q_ is a vector of observations of dichotomized self-reported health in quintile *q* and *X*_q_ is a matrix of observed social determinants and a constant, then the difference between average self-reported health in the lowest quintile, *ȳ*_1_, and the highest quintile, *ȳ*_5_, can be fit using linear regression and broken down into 2 components: first, a component that is attributable to differences in *X*_q_ between the quintiles (ie, differences in their composition) and, second, a component that is attributable to differences in associations between *y*_q_ and *X*_q_, captured by the coefficients in the linear model, β_q_. If we use the associations of the fifth and richest quintile, β_5_, as our benchmark for *best health*, then the estimated difference in average self-reported health between that quintile and the worst-off quintile, β_1_, is decomposable as follows:



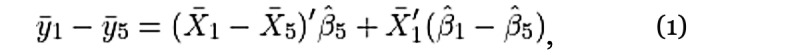



If social determinants in *X*_q_ are indexed by *m*, then the estimated proportional contribution of determinants *m* ∈ *G* is as follows:



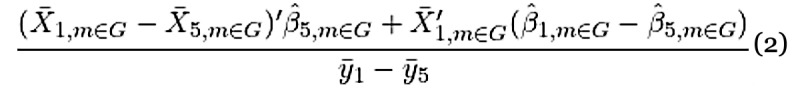



We will estimate equation 2 for each group of categories of social determinants and levels. Our baseline estimates of β_q_ will use pooled multilevel logistic regression with mixed effects and include all predictors in *X*_q_ identified by our nodes (use of logistic regression will slightly modify the aforementioned notation, but the interpretation will remain the same [75,76]).

Data will be pooled to ensure sufficiently large local sample sizes (in previous work [74], we have found that pooling ≥5 years is needed—at this time, we only have 7 years, which is not enough for two 5-year pooled intervals). We will include indicators for year to control for period effects. Provincial effects will be set to fixed, and meso-level variable effects (ie, health region and metropolitan area or municipality) will be set to random. As this is the first study of its kind, we will explore different model specifications and document the implications of these choices for our decomposition results [77,78].

All statistical estimates will be calculated using probability weight, and SEs will be calculated using bootstrapping to account for the complex survey design of the CCHS [79].

### Interaction and Effect Modification Analysis

We will also examine the impacts of accommodating interaction and effect modification [80] between certain predictors—specifically, self-reported gender (ie, male; female; and, when possible, nonbinary) and different categories of non–White-identifying (eg, Black-identifying) individuals—and fitting random effects at lower levels (eg, aggregate dissemination area or census tract). This would allow us to see, for instance, whether the impact of education is more significant for women than for men [81,82].

### Ethical Considerations

The University of Saskatchewan Research Ethics Board deemed this study exempt (E486). All datasets used in this study are covered under existing data sharing agreements within the RDC network and provided to researchers in a controlled research environment. The required data linkages have already been completed by Statistics Canada. Furthermore, the datasets are internally deidentified, harmonized, and thoroughly documented by Statistics Canada and its partners, ensuring secure and consistent use for research purposes. No monetary compensation was paid.

## Results

We have used our bridge funding to hire a research specialist full time to obtain ethics approval and data access, complete environmental scans, and begin assembling the linked individual-level data components that are accessible within the RDC (roughly, the preliminary work proposed for the Hub). A prototype version of CSDUL incorporating a limited set of indicators has been developed in a Statistics Canada RDC. We anticipate receiving the finalized indicators from the nodes by August to September 2025 and aim to complete the decomposition analysis by December 2025. In addition, in the beginning year of the project, the Hub will take primary responsibility for integrating the work and components completed by the nodes in the RDC and creating and disseminating CSDUL. It will also work with the nodes simultaneously to create and validate social and environmental area-based indicators at the macro and meso levels that will be merged with the survey and administrative data. It will also construct individual-level demographic variables, health behavior indicators, and health history and health system use indicators. Individual health behaviors (ie, smoking, drinking, physical activity, and frequency of fruit and vegetable consumption) will be operationalized in the CCHS using established methods developed by Statistics Canada [50]. Demographic variables, including age, gender, ethnicity, marital status, family composition, level of education, family income, employment status, and occupation, will be added. For health history and health system use, Discharge Abstract Database and National Ambulatory Care Reporting System data combined with *International Classification of Diseases* coding will be used to identify comorbidities (ie, the Charlson Comorbidity Index). In the second and third years of the project, the Hub will complete the decomposition of the income-related inequalities in self-reported health.

## Discussion

### Expected Findings

The WHO Commission on Social Determinants of Health has called for a *third wave* in population health research, which “makes explicit that health systems and the people who use them exist within a social context that can powerfully determine people’s chances to be healthy—not only through access to health services, but also through access to a range of other resources, opportunities, and rights” [83]. There are numerous social determinants that can impact health outcomes, and the way in which these factors interact with each other is complex [8,84]. For example, *political and economic context* refers to a broad set of structural and functional aspects of society that cannot be directly measured at the individual level but have an immense influence on the patterns of social stratification and, therefore, on the health of populations [21]. The environmental conditions in which populations live, work, and play have a profound influence on their health and well-being [21]. Exposure to harmful elements of the built environment—pollution, low-quality housing, and low amounts of green space, for example—can differ within cities and across subpopulations, leading to environmentally mediated health disparities in specific conditions and overall health status [85]. Biological factors, which include age and sex, also need to be considered when examining the impact of SDOHs in health inequality research [21]. Material circumstances are the direct factors that determine an individual’s ability to live a healthy life [21]. These factors include income, education, employment status, and food security, among others. These factors are proximate measures of socioeconomic position that provide insights into the level of resources available to individuals to enable health-promoting conditions and behaviors [21]. In contrast, contextual determinants such as the socioeconomic environment provide individuals with the opportunity to live a healthy life [21]. The health system is considered an intermediary SDOH, and improving equity in health care requires addressing not only access but also the appropriateness and acceptability of care. Promoting intersectoral action to enhance these aspects can improve overall health outcomes and reduce differential quality of care [21,86]. The WHO SDOH framework has evolved, drawing on various models developed for different purposes, including public education and advocacy, to better understand the complex interrelationships between social factors. It is important to note that these models, including the aforementioned Commission on Social Determinants of Health, have limitations. For instance, they often fail to account for critical aspects emphasized in Indigenous communities, such as the roles of culture and land. Our work aims to contribute to the ongoing evolution of SDOH frameworks, offering a more comprehensive and inclusive perspective that reflects current research.

CSDUL will be the first of its kind that will bridge the gap between the WHO’s conceptual and action SDOH frameworks in a data-driven way and clarify our understanding of what are the factors most likely to drive health outcomes in Canada (ie, what makes Canadians sick or healthy). We will operationalize the WHO SDOH framework, which considers the health system as a social determinant. In addition, by linking multiple leading databases that provide information at the micro, meso, and macro levels, CSDUL will also help researchers see how the various categories of determinants operate on health at different levels, as conceptualized by the WHO’s separate but complementary framework for action on tackling SDOHs. It will also allow us to explore interaction effects with identifiable groups (including by gender) to understand how social determinants impact their health differently. Moreover, the data and decomposition methods used in our study will allow us to combine or differentiate among associations between social and structural determinants and more effectively link their conceptualization with the levels at which decisions are made and actions are taken.

CSDUL will also provide a framework and data components that can augment existing microsimulation tools such as Statistics Canada’s Population Health Model [87], which are used to forecast the impacts of health policy decisions. Crucially, the development and future maintenance and dissemination of CSDUL will ensure that Canadian researchers are able to collaboratively use, build on, and learn from the work of others in the RDC. Researchers who are new to the RDC, including health system researchers, will no longer have to start from scratch, thus dramatically reducing the costs of conducting population health and health equity analysis using Canada’s linked health and social data.

### Conclusions

Health inequities and inequalities are common in Canadian cities, but they are not equally distributed across various population groups. Due to data limitations, it has been difficult to conduct large-scale comparative research on health disparities in Canadian cities in the past. However, this situation is rapidly changing, and with the introduction of CSDUL, it will be possible to investigate SDOHs at both the individual and area levels simultaneously and seamlessly. Decomposition analysis is a method for understanding what contributes to health inequities by estimating associations among health inequalities, populations, and correlated determinants, such as those that our hub and nodes will identify, and analyzing the relative impact of each. Our work will go beyond the WHO/Europe analysis by providing data-driven relative contributions of individual-level variables, political and economic context, environmental context, socioeconomic context, material circumstances, and health systems.

## Appendix

Multimedia Appendix 1 Peer review report from the Public, Community & Population Health Committee (PH1), Canadian Institutes of Health Research (CIHR).

## Abbreviations

CanSET: Canadian Social Environment Typology
CCHS: Canadian Community Health Survey
CPHS: Canadian Population Health Survey
CSDUL: Canadian Social Determinants Urban Laboratory
RDC: Research Data Centre
SDOH: social determinant of health
UPHN: Urban Public Health Network
WHO/Europe: World Health Organization Regional Office for Europe
WHO: World Health Organization
