# Supplementary material for: Multilevel Estimation of the Relative Impacts of Social Determinants on Income-Related Health Inequalities in Urban Canada: Protocol for the Canadian Social Determinants Urban Laboratory
Source: JMIR Res Protoc. 2025 Nov 28;14:e71929. doi: 10.2196/71929 (PMC12701341; doi:10.2196/71929)
Supplement: Multimedia Appendix 1 [file resprot_v14i1e71929_app1.pdf]

Canadian Institutes of Health Research/Instituts de recherche en santé du Canada

Notice of Recommendation/Avis de recommandation

Application Number/Numéro de la demande: 496922

Committee Code/Code du comité: PH1

Applicants/Candidats: Dr. Cordell (Cory) Oren Neudorf, Mr. Thilina Bandara, Dr. Charles Plante

With/Avec: Dr. D. Dutton, Dr. D. Fuller, Dr. J. Hasselback, Dr. L. Lix, Dr. N. Muhajarine, Ms. G. Notten, Dr. M. Wolfson, Dr. M. Young, Dr. E. de Villa

Institution paid/ Établissement payé: University of Saskatchewan

Title/Titre: Multilevel estimation of the relative impacts of social determinants on income-related health inequalities in urban Canada: Toward a new Canadian Social Determinants Urban Laboratory

Primary Inst./Inst. principal: Population and Public Health

Other Related Inst./ Autres inst. connexes:

Competition /Concours: Project Grant
March/Mars 08, 2023

Number in competition/Nbre de demandes dans le concours: 2113

Peer Review Committee Recommendation, for your information and use/ Recommandation du comité d'examen par les pairs, pour fins d'information et d'utilisation:

**Committee/Comité:**	Public, Community & Population Health
**Number reviewed/ Demandes examinées:**	63
**Application rank within the committee/ Rang de la demande dans le comité:**	7
**Percent Rank within the committee / Rang en pourcentage au sein du comité:**	90.32%
**Rated / Cote:**	4.34
**Recommended Term/ Durée recommandée:**	3 years/ans 6 months/mois
**Recommended average annual operating amount/ Montant annuel moyen recommandé pour le fonctionnement:**	$242,857
**Recommended equipment amount/ Montant recommandé pour les appareils:**	$0

This document is for information only.
An application rated below 3.50 is ineligible for CIHR funding. For applications rated 3.50 and above, please note that it is the application's rank within the peer review committee that determines whether it is funded, rather than its absolute rating. The final funding decision will be communicated in the Notice of Decision.

Document à titre d'information seulement.
Une demande cotée en dessous de 3,5 n'est pas admissible au financement des IRSC. En ce qui a trait aux demandes cotées 3,50 ou plus, veuillez noter que l'on détermine l'attribution des fonds en fonction du classement obtenu au sein du comité d'examen par les pairs plutôt qu'en fonction du classement absolu. La décision finale relative au financement sera communiquée dans l'Avis de décision.

**Review Type / Type d'évaluation:**	Reviewer 1 / Évaluateur 1
**Name of Applicant / Nom du chercheur:**	Neudorf, Cordell (Cory)
**Application No. / Numéro de demande:**	496922
**Agency / Agence:**	CIHR/IRSC
**Competition / Concours:**	Project Grant/Subvention Projet
**Committee / Comité:**	Public, Community & Population Health/Santé publique, santé communautaire et santé des populations
**Title / Titre:**	Multilevel estimation of the relative impacts of social determinants on income-related health inequalities in urban Canada: Toward a new Canadian Social Determinants Urban Laboratory

Adjudication Criteria/Critères de sélection

Initial Score/Cote Initiale: 4.2

Top/Bottom Selection/Groupe supérieur/inférieur

- (●) **Top/Groupe supérieur**
- ( ) **Bottom/Groupe inférieur**

**Review Type / Type d'évaluation:**	Reviewer 1 / Évaluateur 1
**Name of Applicant / Nom du chercheur:**	Neudorf, Cordell (Cory)
**Application No. / Numéro de demande:**	496922
**Agency / Agence:**	CIHR/IRSC
**Competition / Concours:**	Project Grant/Subvention Projet
**Committee / Comité:**	Public, Community & Population Health/Santé publique, santé communautaire et santé des populations
**Title / Titre:**	Multilevel estimation of the relative impacts of social determinants on income-related health inequalities in urban Canada: Toward a new Canadian Social Determinants Urban Laboratory

Summary of Application/Résumé de la demande:

The proposal aims to bring together data sources on the social determinants of health to understand their impacts on health outcomes at different jurisdictional levels in Canada.

The primary aim is to integrate the Canadian Population health Survey with other leading social determinants of health data to:

1)Develop and distribute the world's first fully integrated virtual, multilevel, and longitudinal social determinants laboratory environment, which we will call the Canadian Social Determinants Urban Laboratory, and;

2)Replicate the decomposition analysis of income-related inequalities in self-reported health carried out by the WHO/Europe for urban Canada and report on the relative impact of social determinants.

Strengths and Weaknesses/Forces et faiblesses:

Strengths - Significance and Impact of the Research

The proposal identifies a key gap in data and evidence related to SDOH in Canada, with a clear rationale and feasible roadmap for addressing this gap through better integration SDOH measures from existing data sources in Canada.

Most impactful, would be the SDOH indicators generated by some of the nodes (e.g., housing) that can be used to achieve the stated objectives and if made available for other researchers to examine their impact on population health and health equity (e.g., through HDPR). As such, there is a high potential for impact on SDOH research in Canada.

Weakness - Significance and Impact of the Research

The background could include a more in depth examination existing SDOH and health surveillance in Canada (e.g., PHAC, CIHI) to highlight how CSDUL will fill the gap. For example, CCHS and its linked dataset is commonly used in surveillance and research. Outlining how CSDUL, using CCHS, will resolve missing national standards and accelerate investigations of underserved regions would clarify the projects contribution.
Decomposition analyses using the CCHS data in Canada to examine social inequities in health also exist, but are not referenced. This information would further highlight the potential of this work to advance knowledge.
In Objective 1, it is not clear whether CSDUL is a team (hub and nodes) or the “suite of programs, algorithms, and data components that will run inside Statistics Canada’s RDC”. Specifically, what is a multilevel and longitudinal virtual laboratory environment and how will this be operationalized within the RDCs? For example, is it describing infrastructure (a common project folder at the RDC) or a new data resource (e.g., similar to CanCHEC) that can be requested for research purposes? Or is it the set of new indicators derived by the team (at the area-level) or statistical code to create the indicators that can be linked to data inside and/or outside of the RDC?

The proposal presents detailed methods for bring together various data sources, however, as stated, the objectives could be clarified.

Objective 2 could be restated as a research question(s). The project eludes to examining heterogeneity of effects across subpopulations, including gender, but this is not included in the objectives. Similarly, the introduction and KTE speak to the needs of underserved regions in Canada, but the objectives do not describe if or how this need addressed by the knowledge users will be addressed. As a result, the anticipated contribution of the work is not clearly described, and perhaps understated. Additional description of research productivity from the data resources that is created would help demonstrate the utility of what is being described.

Strengths - Approaches and Methods

The proposal would bring together existing data to fill an important gap in SDOH data. The idea is well described.

The proposal will build off previous work from the investigator teams, bringing together expertise working with the existing data source and SDOH measures. New SDOH indicators will also be created and shared with the broader research community.

The decomposition methodology is well described and appropriate given the described objectives. Although, much of the exposure measurement is cross-sectional in the CPHS and therefore notions that the analyses move towards causal are overstated.

The iKTE approach, including documented work with the UPHN, is a strength of this application.
Weakness - Approaches and Methods

Additional detail on SDOH variable development and validation required to establish CSDUL would be valuable to assess the scope and feasibility of the project and assess its contribution. Many of the indictors are not fully described and validation methods are not described. For example, qualitative indicators of individual health history and system use and the government spending on health and social indicators are mentioned, but methods not described. This applies to the description for each node’s variables, with Node 5 indicators being broadly described. Many of these variables are later named in the predictor variable description.

While the investigators note their familiarity with the redesign of the CCHS over time and that they will deal with it in similar ways as their previous work. Although, these methods are not described. The potential limitations are understated, in particular as questions and optional modules vary across time and geographies in addition to the survey design.

Based on the description, it is not clear whether the results of the decomposition will be reported by cities/towns or whether this is will be done for SDOH indicators or what time frames will be available for the indicators. Will this be possible given the sample size in the CPHS? Elaborating on past work (ref 10) would be helpful in evaluating what is being proposed.

Is there a strategy for managing the predictor variables in the model beyond including all indicators? How will model specifications be tested and evaluated for groups of variables (e.g., from the nodes) and when will differences be reported separately, for example by gender (men and women) or by non-white categories.

Sex and gender considerations are limited.

Minor point, but if variables from health admin data are not available for Quebec, are they excluded from CSDUL?

Expertise, Experience and Resources

The NPA has an excellent track record and leadership in this field. The team is made up of a diverse group of investigators at a mix career stages for each of the nodes being advised by a Senor Advisory Group. The work has a substantial training component, including trainees and early career investigators. However, it was surprising that no doctoral students were included given the breadth of data being consolidated.

**Review Type / Type d'évaluation:**	Reviewer 1 / Évaluateur 1
**Name of Applicant / Nom du chercheur:**	Neudorf, Cordell (Cory)
**Application No. / Numéro de demande:**	496922
**Agency / Agence:**	CIHR/IRSC
**Competition / Concours:**	Project Grant/Subvention Projet
**Committee / Comité:**	Public, Community & Population Health/Santé publique, santé communautaire et santé des populations
**Title / Titre:**	Multilevel estimation of the relative impacts of social determinants on income-related health inequalities in urban Canada: Toward a new Canadian Social Determinants Urban Laboratory

Budget Recommendation/Recommandation budgétaire:

No concerns.

Please indicate your appraisal of the integration of sex as a biological variable as a strength, weakness, or not applicable to the proposal./Prière de sélectionner une option pour donner votre évaluation de l'intégration du sexe comme variable biologique en tant que point fort ou point faible de la proposition, ou en tant qu'élément non applicable à la proposition.

- ○ **Strength/Point fort**
- ○ **Weakness/Point faible**
- ◉ **Not applicable/Non applicable**

Please indicate your appraisal of the integration of gender as a socio-cultural determinant of health as a strength, weakness, or not applicable to the proposal./Prière de sélectionner une option pour donner votre évaluation de l'intégration du genre comme déterminant socioculturel de la santé en tant que point fort ou point faible de la proposition, ou en tant qu'élément non applicable à la proposition.

- ◉ **Strength/Point fort**
- ○ **Weakness/Point faible**
- ○ **Not applicable/Non applicable**

**Review Type / Type d'évaluation:**	Reviewer 1 / Évaluateur 1
**Name of Applicant / Nom du chercheur:**	Neudorf, Cordell (Cory)
**Application No. / Numéro de demande:**	496922
**Agency / Agence:**	CIHR/IRSC
**Competition / Concours:**	Project Grant/Subvention Projet
**Committee / Comité:**	Public, Community & Population Health/Santé publique, santé communautaire et santé des populations
**Title / Titre:**	Multilevel estimation of the relative impacts of social determinants on income-related health inequalities in urban Canada: Toward a new Canadian Social Determinants Urban Laboratory

Sex and/or Gender Considerations/Notions de sexe et/ou de genre:

The interaction between gender and other SDOH will be explored through interactions and sometimes stratified analyses.

These could be more explicitly stated in the objectives.

**Review Type / Type d'évaluation:**	Reviewer 2 / Évaluateur 2
**Name of Applicant / Nom du chercheur:**	Neudorf, Cordell (Cory)
**Application No. / Numéro de demande:**	496922
**Agency / Agence:**	CIHR/IRSC
**Competition / Concours:**	Project Grant/Subvention Projet
**Committee / Comité:**	Public, Community & Population Health/Santé publique, santé communautaire et santé des populations
**Title / Titre:**	Multilevel estimation of the relative impacts of social determinants on income-related health inequalities in urban Canada: Toward a new Canadian Social Determinants Urban Laboratory

Adjudication Criteria/Critères de sélection

Initial Score/Cote Initiale: 4.4

Top/Bottom Selection/Groupe supérieur/inférieur

- (selected) **Top/Groupe supérieur**
- ( ) **Bottom/Groupe inférieur**

**Review Type / Type d'évaluation:**	Reviewer 2 / Évaluateur 2
**Name of Applicant / Nom du chercheur:**	Neudorf, Cordell (Cory)
**Application No. / Numéro de demande:**	496922
**Agency / Agence:**	CIHR/IRSC
**Competition / Concours:**	Project Grant/Subvention Projet
**Committee / Comité:**	Public, Community & Population Health/Santé publique, santé communautaire et santé des populations
**Title / Titre:**	Multilevel estimation of the relative impacts of social determinants on income-related health inequalities in urban Canada: Toward a new Canadian Social Determinants Urban Laboratory

Summary of Application/Résumé de la demande:

Social determinants are the primary driver of the health of Canadians, but the empirical basis for this claim remains thin. For instance, in Europe, as much as 89% of the variation in self-reported health could be explained by some combination of the social determinants of health and, among them, income and social security protection have the greatest impact. Canada has data capable of articulating globally unprecedented multilevel models of the social determinants of health and their impacts on health outcomes at provincial, regional, community, and person levels, but it remains underutilized.

The aims of this project would be to work with Statistics Canada's integrated Canadian Population Health Survey data (CPHS) and other leading social determinant data to:

1)Develop and distribute the world's first fully integrated virtual, multilevel, and longitudinal social determinants laboratory environment (called CSDUL), and

2)Replicate the decomposition analysis of income-related inequalities in self-reported health carried out by the WHO/ Europe for urban Canada and report on the relative impact of social determinants.

The lab will consist of a suite of programs, algorithms, and data components which will assemble information and derive variables from at least 15 leading data sources to support multi-level statistical analysis of how social and non-social factors determine people's health. Five "Nodes", each led by a different early or mid-career investigator, will work with other leading sources of social and environmental data to create and validate area-based indicators at macro and meso levels that will be merged with the survey and administrative data. A central Hub will assemble and disseminate CSDUL. CSDUL will be designed to operate on micro-data within Statistics Canada's Research Data Centres.

This study will provide insight into how limited health resources can be allocated to maximize their impact on population health.

CSDUL, its components, and their validation will be distributed to the wider research community through the existing research data infrastructure of the Health Data Research Network Canada and through peer reviewed academic publications. A fully designed capstone report presenting the results of the decomposition analysis will be prepared in collaboration with the National Collaborating Centre for Healthy Public Policy and disseminated to decision-makers and the public in English and French.

This is a resubmission of a previously unsuccessful application – the team was awarded a bridge grant. The applicants seem to have addressed most previous comments.

**Review Type / Type d'évaluation:**	Reviewer 2 / Évaluateur 2
**Name of Applicant / Nom du chercheur:**	Neudorf, Cordell (Cory)
**Application No. / Numéro de demande:**	496922
**Agency / Agence:**	CIHR/IRSC
**Competition / Concours:**	Project Grant/Subvention Projet
**Committee / Comité:**	Public, Community & Population Health/Santé publique, santé communautaire et santé des populations
**Title / Titre:**	Multilevel estimation of the relative impacts of social determinants on income-related health inequalities in urban Canada: Toward a new Canadian Social Determinants Urban Laboratory

Strengths and Weaknesses/Forces et faiblesses:

STRENGTHS

The proposal is well written and referenced.

The team includes mid-career, new and senior investigators from the University of Saskatchewan, Saskatchewan Health Authority, Dalhousie University, University of Manitoba, University of Ottawa, McMaster University, McGill University, Public Health Agency of Canada, University of Regina, and Concordia University, with knowledge users from Toronto Public Health and the National Public Health Institute of Quebec. It is truly national, and includes a variety of expertise (including medicine, epidemiology, public health, biostatistics, and sociology).

The study is based on strong theoretical models.

This study will inform other projects the team is currently conducting or intend to conduct for which they do not have dedicated funding.

This study will develop an innovative infrastructure which results will be useful to many other research initiatives in which social determinants are studied.

The project would be supported by a postdoctoral fellow and by graduate students (one for the hub and each node, except for Node 1).

The KT plan is strong, with a network of collaborators with Canada’s leading health data stewards and researchers. The project will be advised by both a Knowledge Users Group led by Dr. Eileen de Villa, the lead Medical Officer of Health for the City of Toronto and the Chair of the UPHN, and will include the UPHN membership and Olivier Bellefleur, Scientific and Administrative Lead for the National Collaborating Center on Health and Public Policy (NCCHPP).

The potential for improving decision making as to allocation of resources is important.

WEAKNESSES

The proposal is often written in jargon and has ‘obscure corners’.

The applicants seem to mix up gender and sex in the proposal (ex. pages 7 of 10 and 1 of 2).

**Review Type / Type d'évaluation:**	Reviewer 2 / Évaluateur 2
**Name of Applicant / Nom du chercheur:**	Neudorf, Cordell (Cory)
**Application No. / Numéro de demande:**	496922
**Agency / Agence:**	CIHR/IRSC
**Competition / Concours:**	Project Grant/Subvention Projet
**Committee / Comité:**	Public, Community & Population Health/Santé publique, santé communautaire et santé des populations
**Title / Titre:**	Multilevel estimation of the relative impacts of social determinants on income-related health inequalities in urban Canada: Toward a new Canadian Social Determinants Urban Laboratory

Budget Recommendation/Recommandation budgétaire:

The budget (850 000$ / 3.5 years). This seems appropriate. The bridge grant was subtracted from the original budget.

**Review Type / Type d'évaluation:**	Reviewer 2 / Évaluateur 2
**Name of Applicant / Nom du chercheur:**	Neudorf, Cordell (Cory)
**Application No. / Numéro de demande:**	496922
**Agency / Agence:**	CIHR/IRSC
**Competition / Concours:**	Project Grant/Subvention Projet
**Committee / Comité:**	Public, Community & Population Health/Santé publique, santé communautaire et santé des populations
**Title / Titre:**	Multilevel estimation of the relative impacts of social determinants on income-related health inequalities in urban Canada: Toward a new Canadian Social Determinants Urban Laboratory

Please indicate your appraisal of the integration of sex as a biological variable as a strength, weakness, or not applicable to the proposal./Prière de sélectionner une option pour donner votre évaluation de l'intégration du sexe comme variable biologique en tant que point fort ou point faible de la proposition, ou en tant qu'élément non applicable à la proposition.

- ○ **Strength/Point fort**
- ◉ **Weakness/Point faible**
- ○ **Not applicable/Non applicable**

Please indicate your appraisal of the integration of gender as a socio-cultural determinant of health as a strength, weakness, or not applicable to the proposal./Prière de sélectionner une option pour donner votre évaluation de l'intégration du genre comme déterminant socioculturel de la santé en tant que point fort ou point faible de la proposition, ou en tant qu'élément non applicable à la proposition.

- ◉ **Strength/Point fort**
- ○ **Weakness/Point faible**
- ○ **Not applicable/Non applicable**

Sex and/or Gender Considerations/Notions de sexe et/ou de genre:

The applicants wrote: In this study, the applicants will create a new Canadian Social Determinants Urban Laboratory to investigate how micro, meso, and macro-level determinants impact health. One of the key strengths of this virtual environment is that it will draw on linked surveys and health system administrative data, which means that it will include information on both gender and sex. They will also explore the impacts of interactions between gender and other social determinants. However, in the application, they seem to mix up sex and gender.

**Review Type / Type d'évaluation:**	Reviewer 3 / Évaluateur 3
**Name of Applicant / Nom du chercheur:**	Neudorf, Cordell (Cory)
**Application No. / Numéro de demande:**	496922
**Agency / Agence:**	CIHR/IRSC
**Competition / Concours:**	Project Grant/Subvention Projet
**Committee / Comité:**	Public, Community & Population Health/Santé publique, santé communautaire et santé des populations
**Title / Titre:**	Multilevel estimation of the relative impacts of social determinants on income-related health inequalities in urban Canada: Toward a new Canadian Social Determinants Urban Laboratory

Adjudication Criteria/Critères de sélection

Initial Score/Cote Initiale: 4.4

Top/Bottom Selection/Groupe supérieur/inférieur

- (●) **Top/Groupe supérieur**
- ( ) **Bottom/Groupe inférieur**

**Review Type / Type d'évaluation:**	Reviewer 3 / Évaluateur 3
**Name of Applicant / Nom du chercheur:**	Neudorf, Cordell (Cory)
**Application No. / Numéro de demande:**	496922
**Agency / Agence:**	CIHR/IRSC
**Competition / Concours:**	Project Grant/Subvention Projet
**Committee / Comité:**	Public, Community & Population Health/Santé publique, santé communautaire et santé des populations
**Title / Titre:**	Multilevel estimation of the relative impacts of social determinants on income-related health inequalities in urban Canada: Toward a new Canadian Social Determinants Urban Laboratory

Summary of Application/Résumé de la demande:

The overarching goal of this project is to understand how the social determinants of health (SDoH) at the macro, micro, and meso-level contribute to the health of the Canadian population. The proposal aims to recreate the Europe/WHO estimates of individual contributions of SDoH (e.g., income or access to healthcare) to health outcomes. The specific aims of this project are to, 1) create a virtual laboratory (Canadian Social Determinants Urban Laboratory (CSDUL)) to consolidate and house the available data sources required to examine the impact of SDoH on health outcomes, and 2) replicate the decomposition analyses performed in Europe focused on estimating inequalities in self-reported health associated with individual-level income. The team proposes to address these aims through a pan-Canadian initiative using national data linked across 15 data sources (CCHS, CPHS, CANUE, and publicly available data) stored at the Statistics Canada Research Data Centre. The project will be governed by a hierarchical structure including a main hub and 5 nodes (each addressing a component of the SDoH), with each node responsible for consolidating the data, creating codebooks, and producing valid data elements. Once all data elements have been created, the second aim will serve as a proof of principle for researchers to demonstrate how the data can be utilized for future research focused on the SDoH. The researchers propose to use a decomposition analysis with a multilevel mixed effect models to account for potential between group differences in specific determinants. Interactions between predictors, including gender, will be explored.

**Review Type / Type d'évaluation:**	Reviewer 3 / Évaluateur 3
**Name of Applicant / Nom du chercheur:**	Neudorf, Cordell (Cory)
**Application No. / Numéro de demande:**	496922
**Agency / Agence:**	CIHR/IRSC
**Competition / Concours:**	Project Grant/Subvention Projet
**Committee / Comité:**	Public, Community & Population Health/Santé publique, santé communautaire et santé des populations
**Title / Titre:**	Multilevel estimation of the relative impacts of social determinants on income-related health inequalities in urban Canada: Toward a new Canadian Social Determinants Urban Laboratory

Strengths and Weaknesses/Forces et faiblesses:

Strengths:

• Pan-Canadian team with complimentary expertise including an NPA who is a mid-career investigator with numerous years of experience in health inequities research as the Medical Officer of Health for Saskatchewan Health Authority and the President of the Urban Public Health Network (UPHN). They also have a notable funding record, primarily as NPA. The team includes researchers, public health practitioners, stakeholders at various career stages.

• The team has integrated numerous career development and training opportunities for early career investigators and trainees.

• The rationale for the study was well-justified and grounded in established theoretical frameworks. The rigorous governance plan and well-defined roles/responsibilities of the research team is a strength of the proposal.

• Despite the challenge in implementing the virtual laboratory, the inclusion of experts in the use of administrative health data supports the feasibility of the proposed study. The inclusion of an Advisory Board to provide oversight throughout the process is a strength.

• The impact of this proposal goes beyond the creation of a virtual laboratory. This will provide an important resource for future research to identify the contribution of SDoH to health.

• Well-established network of stakeholders at both the provincial and federal-level and involvement of the NPA and co-PI in KT efforts related to the project to date is an additional strength.

• The team adequately addressed previous reviewers’ comments in the resubmission.

Weakness:

• Further discussion on potential mitigation strategies to overcome any challenges in defining/operationalizing variables could have been added. This challenge is briefly mentioned but could have been expanded on in greater detail.

**Review Type / Type d'évaluation:**	Reviewer 3 / Évaluateur 3
**Name of Applicant / Nom du chercheur:**	Neudorf, Cordell (Cory)
**Application No. / Numéro de demande:**	496922
**Agency / Agence:**	CIHR/IRSC
**Competition / Concours:**	Project Grant/Subvention Projet
**Committee / Comité:**	Public, Community & Population Health/Santé publique, santé communautaire et santé des populations
**Title / Titre:**	Multilevel estimation of the relative impacts of social determinants on income-related health inequalities in urban Canada: Toward a new Canadian Social Determinants Urban Laboratory

Budget Recommendation/Recommandation budgétaire:

The majority of the budget is allocated to support research staff and trainees, which seems reasonable given the scope of the proposed project. No budget or ethical concerns to flag.

Please indicate your appraisal of the integration of sex as a biological variable as a strength, weakness, or not applicable to the proposal./Prière de sélectionner une option pour donner votre évaluation de l'intégration du sexe comme variable biologique en tant que point fort ou point faible de la proposition, ou en tant qu'élément non applicable à la proposition.

- (●) **Strength/Point fort**
- ( ) **Weakness/Point faible**
- ( ) **Not applicable/Non applicable**

Please indicate your appraisal of the integration of gender as a socio-cultural determinant of health as a strength, weakness, or not applicable to the proposal./Prière de sélectionner une option pour donner votre évaluation de l'intégration du genre comme déterminant socioculturel de la santé en tant que point fort ou point faible de la proposition, ou en tant qu'élément non applicable à la proposition.

- (●) **Strength/Point fort**
- ( ) **Weakness/Point faible**
- ( ) **Not applicable/Non applicable**

**Review Type / Type d'évaluation:**	Reviewer 3 / Évaluateur 3
**Name of Applicant / Nom du chercheur:**	Neudorf, Cordell (Cory)
**Application No. / Numéro de demande:**	496922
**Agency / Agence:**	CIHR/IRSC
**Competition / Concours:**	Project Grant/Subvention Projet
**Committee / Comité:**	Public, Community & Population Health/Santé publique, santé communautaire et santé des populations
**Title / Titre:**	Multilevel estimation of the relative impacts of social determinants on income-related health inequalities in urban Canada: Toward a new Canadian Social Determinants Urban Laboratory

Sex and/or Gender Considerations/Notions de sexe et/ou de genre:

The team will explore potential differences in self-reported health by gender and sex in Aim 2. There was some confusion with the terms sex and gender in the proposal.

Review Type/Type d'évaluation: SO Notes /Notes de l'agent scientifique
Name of Applicant/Nom du chercheur: Neudorf, Cordell (Cory) Oren
Application No./Numéro de demande: 496922
Agency/Agence: CIHR/IRSC
Competition/Concours: 2023-03-08 Project Grant/Subvention Projet
Committee/Comité: Public, Community & Population Health/Santé publique, santé communautaire et santé des populations
Title/Titre: Multilevel estimation of the relative impacts of social determinants on income-related health inequalities in urban Canada: Toward a new Canadian Social Determinants Urban Laboratory

---

Assessment/Évaluation:

Strengths (including SGBA considerations):

- This is a strong infrastructure building study
- Diverse group of investigators at different career stages, with the nodes of research led by early and mid-career researchers supported by senior investigators
- Very good iKT plan working with relevant networks
- Good sharing of the data through the health data network
- Good integration of datasets that will enable addressing knowledge gaps, underpinned using the leading conceptual framework in the field

Weaknesses (including SGBA considerations):

- The background provided only a cursory review of the available datasets and approaches
- There was a lack of clarity around the research lab environment and how other researchers will access this lab and resource – particularly within the RDC environment and limitations for access
- Limited research questions were identified by study investigators, whereas this infrastructure has potential to support many research questions. This was considered an opportunity to define clearer research questions.
- Methods were under described in some areas – for example, variable development was not always that clear, there was concern that mitigation strategies for missing data were not well described, and issues were unidentified around harmonization of the data – e.g., regarding missing data - when Quebec is missing some datasets will they be excluded from elsewhere?
- There was a mixed approach to sex and gender, and these terms were used interchangeably – i.e., how will they get gender from administrative databases?
-

Budget:

- No concerns
